# Editorial: Innovative strategies for enhancing crop resilience against plant viral diseases

**DOI:** 10.3389/fpls.2025.1603589

**Published:** 2025-04-23

**Authors:** Chellappan Padmanabhan

**Affiliations:** United States Department of Agriculture, Animal Plant Health Inspection Service, Plant Protection and Quarantine, Science and Technology, Plant Pathogen Confirmatory Diagnostics Laboratory, Laurel, MD, United States

**Keywords:** artificial intelligence, meta-transcriptomes, virus-pathogenesis, virus-suppressors of silencing, vector transmission, seed pathology, disease resistance

This Research Topic covers various subjects related to virus disease detection and translates into plant protection against viruses.

## Artificial intelligence

AI for pathogen detection is still in its early stages, and two research articles utilizing the YOLOv4 and PDLM-TK algorithms elegantly described AI-based pathogen detection of plant leaf diseases. To enhance disease prediction systems in agriculture, a large number of images of healthy and diseased leaves of 14 species from the Plant Village dataset were utilized. The YOLOv4 demonstrated high performance with high accuracy on the Plant Village Dataset, including precision, recall, and F1-score. These findings showed that YOLOv4 is an effective tool for accurate disease identification, highlighting significant advances in plant disease detection (Aldakheel et al.). The other article focused on the plant pest and disease lightweight identification model by fusing tensor features and knowledge distillation (PDLM-TK), which represents a further advance in pathogen diagnosis, explicitly addressing the efficiency and accuracy associated with pest and disease identification. This model uses lightweight residual blocks based on spatial tensor (LRB-ST) to obtain enhanced plant image features with a streamlined depth-separable convolution approach with reduced parameter numbers. These results establish PDLM-TK as an alternative to lightweight methods such as MobileViT, providing high efficiency for detecting plant diseases (Zhang et al.).

## Meta-transcriptomes

One of the research articles reported on the application of the meta-transcriptome approach for the identification of a virome associated with sugar beets. In addition to detecting known viruses infecting sugar beets, this study identified the spread of the beta vulgaris satellite virus in new locations, indicating its geographical expansion. In addition, a novel virus, the Erysiphe necator-associated abispo virus, was identified across all libraries and found to originate from different sugar beet growing locations in the United States (Chinnadurai et al.).

## Host-susceptibility factors

Understanding the interaction between viral infection and plant cellular mechanisms highlighted the role of the Kunitz peptidase inhibitor-like protein (KPILP) in regulating chloroplast retrograde signaling and facilitating the transport of essential macromolecules during viral infection. Research indicates that viruses such as the Tobacco mosaic virus (TMV) and the crucifer-infecting tobamovirus (crTMV) exploit host factors to enhance their replication and spread by suppressing the plant’s defense mechanisms. The results of this study showed reduced levels of KPILP mRNA in systemic infections, indicating its importance for viral survival. The role of the KPILP gene in virus resistance was demonstrated by the fact that the silencing of this gene affected the transport efficiency of both TMV and crTMV. This research provides further strategies for the development of virus control measures for TMV (Ershova et al.).

## Virus-pathogenesis

Currently, the *Tomato leaf curl New Delhi virus* (ToLCNDV) is recognized as a significant viral pathogen and poses a threat to tomato, pepper, and cucurbit crops across the world. In this study, coat protein swapping identified a single amino acid in the coat protein coding sequence of ToLCNDV as the pathogenicity factor associated with virus infection in tomato. This research identified a critical molecular factor that can be used for future breeding for resistance against ToLCNDV (Vo et al.).

## Virus-suppressors of silencing

The strawberry mottle mosaic virus (SMoV) impacts strawberry productivity. In this article, the authors identified two silencing suppressors, Pro2Glu and P28, from SMoV. Furthermore, Pro2Glu and P28 were found to play a role in increasing the accumulation of potato virus X. This study has implications for formulating strategies to control viral diseases in strawberry and other crops (Fan et al.).

## Vector transmission

One of the articles discussed the prevalence and transmission dynamics of the black raspberry necrosis virus (BRNV) and other viruses affecting raspberry in Norway based on three years of data. Infection rates showed that the old raspberry cultivar Veten, including wild raspberry populations, is susceptible to BRNV. Additionally, the known aphid vector, Amphorophora aidaei, is able to acquire the virus within one minute, and transmission can occur within one hour. Understanding the mechanisms behind virus transmission is essential for protecting raspberry crops from these ongoing agricultural threats (Sapkota et al.).

## Seed pathology

A study focusing on seed transmission and elimination strategies investigated how viral infections impact the alfalfa (Medicago sativa) industry, specifically targeting six significant viruses: Alfalfa mosaic virus (AMV), Medicago sativa alphapartitivirus1 (MsAPV1), MsAPV2, Medicago sativa deltapartitivirus 1 (MsDPV1), amalgavirus 1(MsAV1), and Cnidium vein yellowing virus 1 (CnVYV1). Virus transmission rates from alfalfa seeds to seedlings were approximately 44% to 88% using PCR assay. The authors tested 16 virus elimination strategies for alfalfa seeds. In conclusion, this research sheds light on critical virus transmission pathways in alfalfa, offers practical methods for managing these diseases, and enhances agricultural productivity (Li et al.).

## Disease resistance

Cassava (Manihot esculenta Crantz) is a stable food crop in many countries and has potential as a biodegradable material. In Africa, viral diseases originating from the cassava brown streak virus (CBSV) and the cassava mosaic virus (CMD) challenge cassava production. In this article, quantitative trait loci (QTLs) for CBSD resistance were identified. Two important QTLs on chromosome 4 were linked to resistance against CBSD foliar symptoms, while another QTL on chromosome 11 was linked to root necrosis. The study emphasized key candidate genes, such as phenylalanine ammonia-lyase (PAL) and cinnamoyl-CoA reductase (CCR), which play an essential role in the lignin biosynthesis pathway—critical for plant defense mechanisms against pathogens. This study provides the foundation for breeding strategies to cope with future challenges against viral diseases (Ferguson et al.).

## Biocontrol

Biocontrol is important as it is an efficient and safe way to control plant diseases. Pepper veinal mottle virus (PVMV) is a concern for pepper growers. In this study, Bacillus velezensis HN-2, was shown to delay the PVMV infection by its ability colonizing in the intercellular spaces of plants, and induction of the host defense, specifically Jasmonic acid pathway. Accordingly, B. velezensis HN-2 has the potential to provide alternative strategy for virus control (Xuan et al.).

Two review articles focused on virus disease detection and plant protection against viruses. The first article discussed recent advances in diagnostic technologies and their challenges, in addition to conventional methods such as enzyme-linked immunosorbent assay, polymerase chain reaction and isothermal amplification and biosensor technologies. Sequencing-based diagnostics include next-generation sequencing and portable nanopore sequencing, and CRISPR-Cas assays. These technologies enable faster and more precise detection of plant pathogens (Kanapiya et al.). The other review article focused on plant protection strategies and challenges (Anikina et al.).

In conclusion, all the contributions to this Research Topic highlight the timely translation of plant disease management into major applications.

